# Sports activity participation after curved periacetabular osteotomy for acetabular dysplasia

**DOI:** 10.1186/s12891-020-03625-3

**Published:** 2020-09-28

**Authors:** Yoshiki Takahashi, Naonobu Takahira, Katsufumi Uchiyama, Kensuke Fukushima, Mitsutoshi Moriya, Manaka Shibuya, Kouji Tsuda, Kiyoshi Tozaki, Sho Kudo, Hiroaki Kaneda, Junya Sekita, Masashi Takaso

**Affiliations:** 1 Sensory and Motor Control, Graduate School of Medical Sciences, Kitasato University, 1-15-1 Kitasato, Minami-ku, Sagamihara-city, Kanagawa 252-0373 Japan; 2 Department of Orthopaedic Surgery of Clinical Medicine, Rehabilitation Sciences and Functional Restoration, Science of Sensory and Motor Control, Graduate School of Medical Sciences, Kitasato University, 1-15-1 Kitasato, Minami-ku, Sagamihara-city, Kanagawa 252-0373 Japan; 3 Department of Rehabilitation, School of Allied Health Sciences, Kitasato University, 1-15-1 Kitasato, Minami-ku, Sagamihara-city, Kanagawa 252-0373 Japan; 4 Department of Orthopaedic Surgery, School of Medicine, Kitasato University, 1-15-1 Kitasato, Minami-ku, Sagamihara-city, Kanagawa 252-0374 Japan; 5 Department of Rehabilitation, Kitasato University Hospital, 1-15-1 Kitasato, Minami-ku, Sagamihara-city, Kanagawa 252-0375 Japan

**Keywords:** Curved periacetabular osteotomy, Sports activity, Acetabular dysplasia, Physical function

## Abstract

**Background:**

Curved periacetabular osteotomy (CPO) was developed to treat acetabular dysplasia. Given that CPO can improve physical function in the early post-operative period, patients might be able to participate in sports activities post-operatively. Therefore, this study examined the post-operative sports activity participation and characteristics of acetabular dysplasia patients who have undergone CPO.

**Methods:**

A total of 52 patients who underwent CPO for acetabular dysplasia were given a questionnaire on pre- and post-operative sports activities; 43 patients responded. We surveyed patients’ sports activities, satisfaction, and physical function. Patients were divided according to whether they participated in sports activities after CPO. Physical function was compared before and after CPO.

**Results:**

The pre- and post-operative sports activity participation rates were 55.8 and 72.1%, respectively. Patients mostly performed low-impact sports activities. Moreover, patients who participated in sports activities post-operatively had smaller pre-operative range of motion of hip flexion and returned to full weight bearing earlier.

**Conclusions:**

Among acetabular dysplasia patients who underwent CPO, 72.1% participated in sports activities post-operatively. Post-operatively, patients participated not only in low-impact sports activities, but also in high-impact ones. These findings might be useful for advising patients who are concerned about participating in sports activities after CPO.

## Background

Acetabular dysplasia is the most common cause of secondary osteoarthritis (OA) [1]. Morphological features due to acetabular dysplasia result in instability and abnormal loading on the articular cartilage [2, 3]; this can lead to degeneration of the articular cartilage, which in turn leads to OA. OA occurs in 15.7% of patients [4], causing pain and limiting range of motion, which can disturb participation in sports activities. Periacetabular osteotomy (PAO) is beneficial for young patients with acetabular dysplasia, with case series showing improvement in post-operative physical activity levels [5, 6] while preserving 60% of hips without requiring total hip arthroplasty at 20 years [7]. Moreover, van Bergayk et al. report that 47.6 and 95.2% of patients with acetabular dysplasia participated in sports activities before and after PAO, respectively [8]. However, another study reports a sports activity participation rate of 55.3% after PAO and a non-significant relationship with OA progression [9].

Meanwhile, curved periacetabular osteotomy (CPO), a modified PAO developed by Naito et al. [10], is indicated for patients with mild or severe dysplastic hips [11]. CPO provides pain relief and early improvement of hip abductor muscle strength because it preserves the hip abductor muscle, which reduces the dynamic instability of the hip joint during walking [12–14]. Another advantage of CPO is its low risk of necrosis of the femoral head and acetabulum owing to the maintenance of blood supply to the rotated acetabulum and the small skin incision required [10]. Thus, these benefits of CPO ultimately reduce post-operative complications, promote early rehabilitation, and increase the likelihood of being able to participate in sports activities. Nevertheless, there are few reports of post-operative daily life in patients who have undergone CPO. Thus, sports activity participation after CPO remains unknown. Given that CPO is a modified PAO, we hypothesised that CPO increases participation in sports activities in patients with acetabular dysplasia to an extent equal to or greater than that of PAO. Therefore, this study evaluated the post-operative sports activity participation and characteristics of patients with acetabular dysplasia who underwent CPO.

## Methods

### Study design and patients

**Fig. 1:**
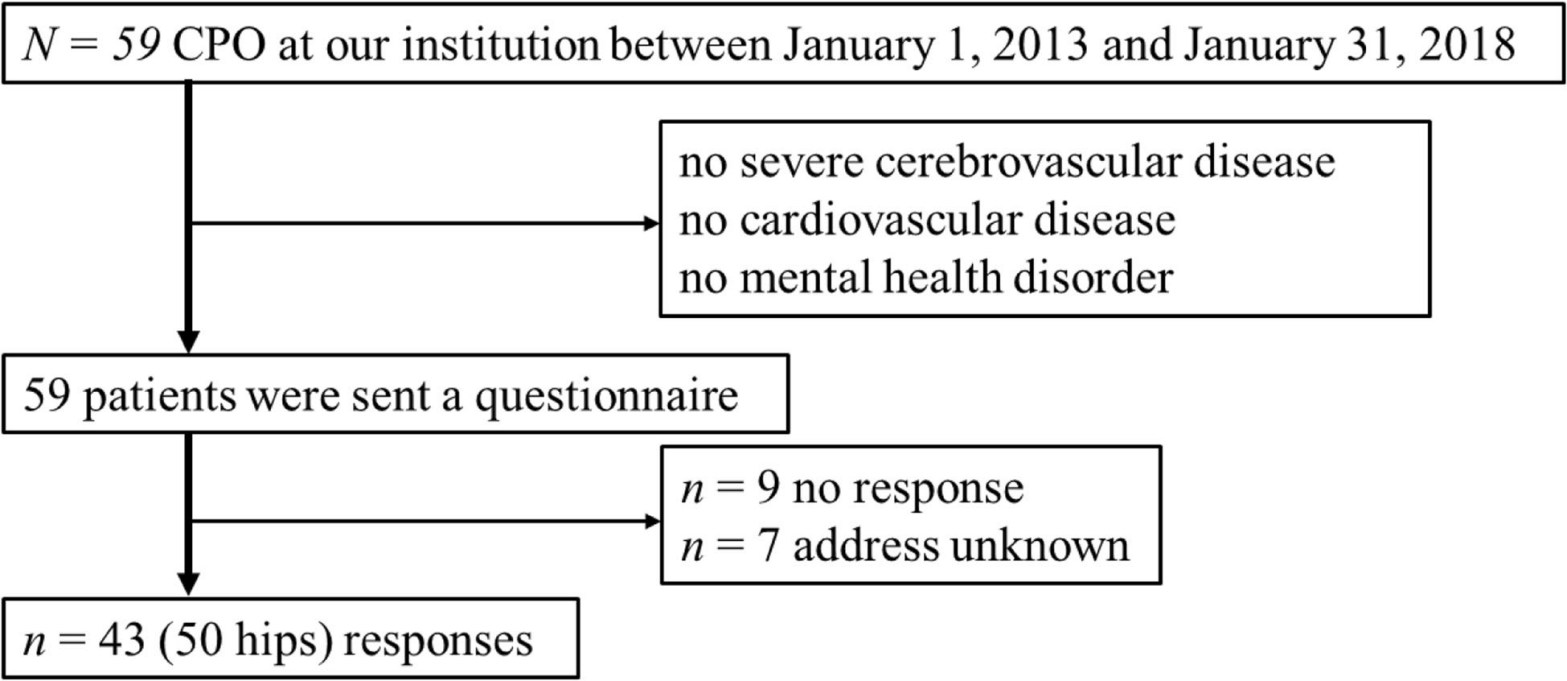
Patient enrolment flowchart. CPO, curved periacetabular osteotomy

This retrospective, questionnaire-based study enrolled 59 consecutive patients with acetabular dysplasia who underwent CPO at our institution between January 1, 2013 and January 31, 2018. We pre-tested the sports activity questionnaire on members of our laboratory and then sent it to patients; 43 patients (82.7%) responded (Fig. 1). Patients with severe cerebrovascular disease, cardiovascular disease, or mental health disorders that limited mobility were excluded.

This study was approved by the ethics committee of our institutions and was conducted in accordance with the Principles of the Declaration of Helsinki and the Good Clinical Practice guidelines. Information about informed consent to participate in the study was enclosed with the questionnaire. An opt-out form for informed consent was adopted.

### Questionnaire

Fifty-two patients were mailed paper questionnaires comprising three sections (Additional file 1). The first section surveyed pre-operative participation in sports activities. Patients were asked whether they participated in sports activities as well as the types and frequency of sports activities. Sports activity participation was defined as exercise that the patient voluntarily performed, and impact was categorised as described by Klein et al. [15]. The second section surveyed patients’ post-operative sports activity participation. Patients who participated in any sports activity were asked why they did so, whereas patients who did not were also asked this question and whether they hoped to participate in sports activities in the future. The third section collected information about patients’ satisfaction with daily activities and sports activities as well as the Forgotten Joint Score (FJS) [16].

### Patient information

Demographic characteristics during hospitalisation were obtained from medical records, including age at the time of surgery, sex, and body mass index (BMI). The following information was also obtained from medical records: time until straight leg-raising exercise possible; time until full weight bearing (FWB) was allowed; post-operative Japan Orthopaedic Association Hip Disease Evaluation Questionnaire (JHEQ) score [17]; and pre-operative clinical characteristics upon admission, including hospitalisation, range of motion, lower-leg muscle strength, 10-m comfortable gait speed, 10-m maximum gait speed, and Harris Hip Score (HHS) [18]. The time until FWB was allowed and post-operative JHEQ scores were obtained at post-discharge follow-up. Range of motion, lower-leg muscle strength, 10-m comfortable gait speed, 10-m maximum gait speed were assessed by physical therapists. The JHEQ comprises hip joint condition (visual analogue scale) and three subscales: pain, movement, and mental. Higher scores indicate higher quality of life.

### Statistical analysis

The data were summarised using descriptive statistics. Pre- and post-operative sports activity participation rates were calculated by dividing the numbers of patients who participated in sports activities pre- or post-operatively by 43 (i.e. the number of patients who responded to the questionnaire). Patients were categorised into the ‘participation group’ or ‘non-participation group’ according to whether they participated in sports activities post-operatively. The Mann-Whitney *U*-test or Kruskal-Wallis test was used, where appropriate, to compare demographic characteristics between groups. All statistical analyses were performed using R version 3.3.3 (R Foundation). *P*-values less than 0.05 were considered statistically significant.

## Results

### Questionnaire

**Fig. 2:**
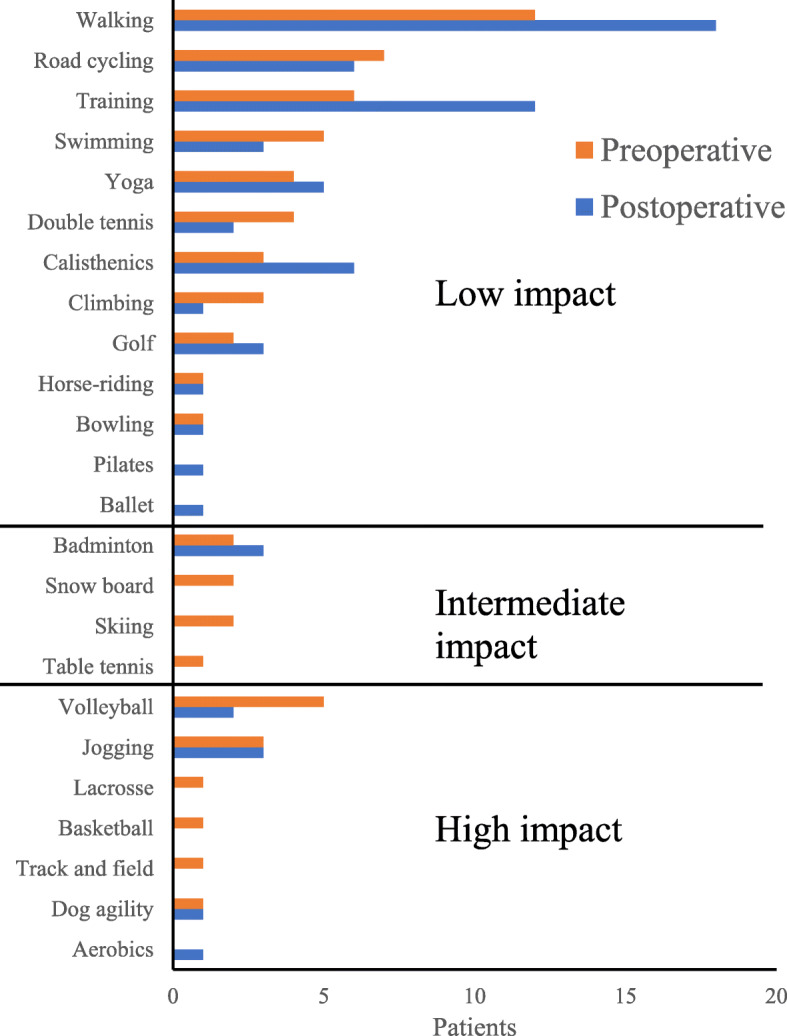
Types and numbers of patients who participated in sports activities before and after curved periacetabular osteotomy (multiple responses allowed)

**Fig. 3:**
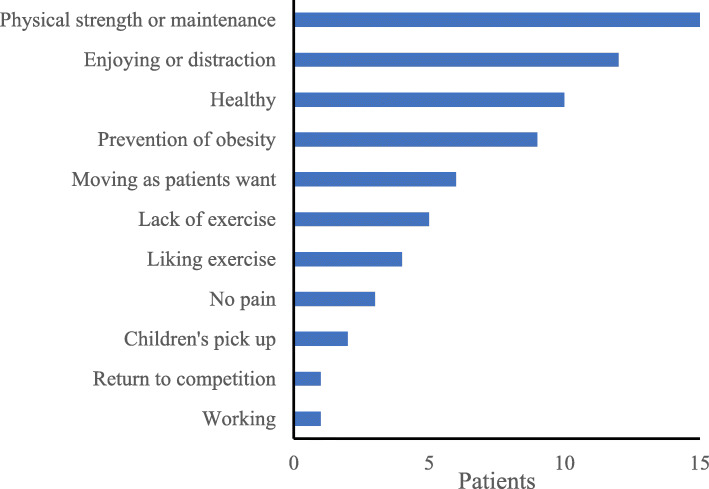
Reasons for participating in sports activities after curved periacetabular osteotomy (multiple responses allowed)

**Fig. 4:**
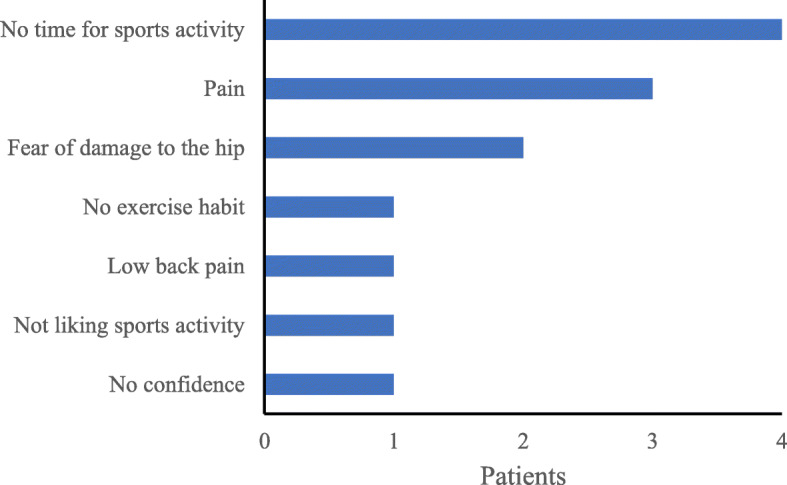
Reasons for not participating in sports activities after curved periacetabular osteotomy (multiple responses allowed)

Patients responded to the questionnaire at a mean of 34.1 ± 17.2 months post-operatively (range: 8–69 months). The pre- and post-operative sports activity participation rates were 55.8% (*n* = 24 patients, 27 hips) and 72.1% (*n* = 31 patients, 36 hips), respectively. The number of patients who participated in sports activities increased significantly from 24 pre-operatively to 31 post-operatively (*P* < 0.01). Of the 24 patients who participated in sports activities pre-operatively, 91.7% (*n* = 22 patients, 24 hips) participated in sports activities post-operatively. At the time of the survey, OA progressed in 1 case (with 46 months of follow-up), but there was no significant association with sports activity participation (*P* = 0.28); this patient did not participate in sports activities pre- or post-operatively. Patients performed many low-impact sports activities such as walking and swimming. Pre-operatively, 10 patients (12 cases) performed high-impact sports activities, such as volleyball and badminton, compared to 7 patients (7 cases) post-operatively (Fig. 2). Among the 7 patients who only participated in sports activities post-operatively, 4 patients participated in walking, 3 participated in training, 3 participated in yoga, 2 participated in road cycling, 2 participated in jogging, 1 participated in swimming, 1 participated in golf, 1 participated in bowling, and 1 participated in aerobics (including duplicate answers). Patients resumed sports activities at a mean of 12.7 ± 10.8 months post-operatively. The mean weekly frequencies of sport activities pre- and post-operatively were 2.7 ± 1.7 and 2.7 ± 2.2 times, respectively. Patients’ reasons for participating or not participating in sports activities are shown in Figs. 3 and 4, respectively.

### Physical function and satisfaction

Physical function and satisfaction were compared between the participation and non-participation groups (Table 1). Compared to the non-participation group, the participation group had a significantly smaller range of motion of hip flexion pre-operatively and significantly earlier post-operative FWB allowed by clinician (*P* = 0.03 and 0.02, respectively). Moreover, 74.1 and 38.8% of patients reported being ‘very satisfied’ or ‘slightly satisfied’ with current life and sports activities, respectively (Table 2).

**Table 1:** Physical function and satisfaction in patients who participated or did not participate in sports activities post-operatively

Factors	Participation group (*n* = 31)	Non-participation group (*n* = 12)	*P*-value
Age (years)	45.0 (42.0–48.5)	48.5 (22.0–58.0)	.61
Body mass index (kg/m^2^)	21.7 (20.8–24.4)	20.7 (20.1–24.5)	.47
Centre–edge angle (°)	11.8 (7.0–15.0)	15.0 (13.8–16.6)	.07
Hospitalization days (days)	39.0 (36.5–43.0)	40.0 (37.8–42.5)	.51
SLR possible (days)	20.0 (15.5–27.0)	20.0 (19.0–22.0)	.91
One-third partial weight bearing allowed (days)	21.0 (21.0–21.0)	21.0 (21.0–21.0)	.77
Full weight bearing allowed (weeks)	13.4 (11.0–14.9)	14.6 (13.9–17.1)	.02
Hip abductor muscle strength (% body weight)			
Operative side	19.2 (12.4–23.2)	22.8 (19.8–24.3)	.16
Non-operative side	19.9 (16.9–26.3)	23.9 (22.9–26.0)	.05
Knee extensor muscle strength (% body weight)			
Operative side	38.6 (26.2–49.2)	42.2 (36.5–51.4)	.60
Non-operative side	41.6 (25.5–51.2)	48.2 (44.2–51.7)	.16
Pain on operative side (VAS, mm)			
Rest	13.5 (0.0–42.0)	15.5 (2.0–28.8)	.91
Gait	48.5 (25.5–80.3)	48.5 (30.5–53.5)	.93
Operative side range of motion (°)			
Hip extension	12.0 (5.0–15.5)	15.0 (11.3–18.8)	.21
Hip flexion	110.0 (93.8–115.5)	117.5 (115.0–120.0)	.03
Hip abduction	30.0 (24.8–35.0)	30.0 (26.3–33.8)	.83
Hip adduction	14.5 (10.0–15.0)	15.0 (10.0–18.8)	.77
Hip external rotation	33.5 (16.8–40.3)	35.0 (27.5–43.8)	.24
Hip internal rotation	31.0 (21.8–41.3)	45.0 (31.8–48.8)	.21
Comfortable gait speed (m/s)	1.1 (1.0–1.2)	1.2 (1.0–1.3)	.65
Maximum gait speed (m/s)	1.7 (1.5–1.8)	1.6 (1.5–1.7)	.84
Harris Hip Score (points)	42.0 (34.0–63.5)	40.0 (31.8–57.3)	.60
Post-operative time at questionnaire completion (months)	30.0 (18.0–48.0)	43.0 (22.3–45.8)	.71
JHEQ Score (points)			
Total	62.5 (45.0–73.8)	65.5 (58.3–81.0)	.27
Pain	22.0 (22.0–26.0)	25.5 (23.5–27.3)	.08
Movement	19.0 (11.5–26.0)	22.5 (16.8–26.5)	.29
Mental	21.0 (14.0–25.0)	20.0 (16.0–28.0)	.73
Hip joint condition (VAS, mm)	12.5 (2.0–19.8)	13.0 (6.9–21.8)	.70
Forgotten Joint Score (points)	60.4 (50.5–75.0)	74.0 (68.8–79.2)	.08

Values are expressed as median (1st–3rd quartile range). *SLR* Straight leg-raising exercise, *VAS* Visual analogue scale, *JHEQ* Japanese Orthopaedic Association Hip-Disease Evaluation Questionnaire

**Table 2:** Satisfaction with daily life and sports activities at the time of the questionnaire

	Daily life	Sports activities
Very satisfied (%)	22.5	19.4
Slightly satisfied (%)	51.6	19.4
Neither (%)	9.7	38.7
Slightly dissatisfied (%)	6.5	9.6
Extremely dissatisfied (%)	9.7	12.9

## Discussion

We investigated acetabular dysplasia patients’ participation in sports activities before and after CPO, and compared patients who participated and did not participate in sports activities post-operatively. Patients who participated in sports activities post-operatively were allowed to return to FWB earlier than those who did not. To our knowledge, this is the first study on the sports activities of patients with acetabular dysplasia who have undergone CPO. Our results will help medical staff ascertain the likelihood that patients with acetabular dysplasia will be able to participate in sports activities after CPO.

In this study, the pre- and post-operative sports activity participation rates of acetabular dysplasia patients who underwent CPO were 55.8 and 72.1%, respectively, versus 31.1 and 55.3% in patients who underwent PAO, respectively [9]. Patients who underwent CPO had significantly higher pre- and post-operative sports activity participation rates than patients who underwent PAO (*P* = 0.047 and = 0.003, respectively). Although these results suggest that CPO might increase post-operative sports activity participation relative to PAO, the pre-operative participation rates were substantially different between studies. Therefore, future studies are required to compare sports activity participation rates after CPO and other osteotomies, such as rotational acetabular osteotomy and PAO, in patients with similar baseline sports activities participation.

Compared to PAO, CPO is less invasive and can create better contact between bone surfaces, resulting in stable fixation and tight bonding [10]. Because early post-operative rehabilitation is possible with CPO, early physical function can be improved. Thus, patients with improved physical function are more willing or able to maintain or improve physical function, resulting in higher sports activity participation. This is corroborated by patients’ self-reported reasons for participating in sports activities (Fig. 2). Furthermore, activity level and satisfaction are reported to be correlated in patients with hip disease [19]. In this study, 74.1% of patients were satisfied with daily life, suggesting that high satisfaction might positively affect sports activity participation. Therefore, medical staff can use the results of this study to provide guidance to patients who want to participate in sports activities after CPO.

Patients participated in sports activities ranging from low to high impact, with the latter being performed less frequently; this trend is similar to previous reports on sports activities before and after total hip arthroplasty [20] or PAO [9]. Many low-impact sports activities are safe for patients to do alone and do not require higher physical functioning in comparison to high-impact sports activities. In addition, post-operative participation in sports activities is reported to be unrelated to OA progression regardless of sports activity impact [9]. Therefore, clinicians can recommend that patients who have undergone CPO participate in low-impact sports activities post-operatively. Meanwhile, a few patients in this study participated in high-impact sports activities. As such activities require high physical functioning, most patients likely refrained from participating in them owing to concerns of deterioration of physical condition, such as OA progression. However, Hara et al. report that among 162 acetabular dysplasia patients, participation in high-impact sports activities after PAO did not significantly influence the progression of OA grade [9]. Because CPO is a modified PAO [10], it is expected to yield the same prognosis as PAO. Hence, some patients participate in high-impact sports activities after CPO or PAO. Therefore, clinicians should support patients who wish to participate in high-impact sports activities with careful follow-up, such as confirmation of OA grade.

The main reasons for not participating in sports activities post-operatively were lack of time and pain. The oldest patient in this study was 62 years. The retirement age in many workplaces in Japan is 65 years. Therefore, given that most patients in this study were younger, many might have had limited time for sports activities because of work. In addition, fear of pain or hip damage hinders post-operative participation in sports activities [21]. Therefore, clinicians should provide patients appropriate guidance for participating in sports activities after obtaining informed consent. Moreover, from the viewpoint of extending healthy life expectancy, it is preferable to advise low-impact sports activities such as walking and cycling. Accordingly, clinicians should avoid giving all patients uniform guidance regarding exercise and instead give individualised guidance, because patients who have undergone CPO might only be able to perform limited sports activities owing to living environment-related or psychological reasons.

In this study, patients who participated in sports activities had a smaller pre-operative hip flexion range of motion than those who did not participate in sports activities. Patients with acetabular dysplasia typically have increased hip flexion, which is reduced by PAO [22]. In other words, patients who participated in sports activities after CPO in this study likely had a smaller flexion angle compared to those who did not participate. Therefore, patients with acetabular dysplasia might be able to participate in post-operative sports activities if they have close-to-smaller hip flexion. In addition, FWB was allowed earlier among patients who participated in sports activities than those who did not, likely because early recovery of muscle strength is associated with early permission for FWB after osteotomy [23]. However, given that allowing FWB when bone fusion is insufficient increases the risk of fracture [24], safety must be prioritised over allowing FWB at an early stage regardless of individual characteristics.

One limitation of our study is its retrospective design using a self-reported questionnaire. Although recall bias might have influenced the results, the recall period was limited to 3 years pre-operatively to minimise such bias. Another limitation is lack of data, specifically post-operative physical function assessments. Although we examined pre-operative physical function and post-operative sports activity, if patients had a certain level of pre-operative physical function, pre-operative physical function might not significantly affect post-operative participation in sports activities. Therefore, further studies examining the effects of post-operative physical function and sports activity participation are needed. Another limitation is the small sample size: few patients were eligible for CPO, especially considering the five-year study period. Therefore, multivariate analysis could not be performed.

## Conclusion

Among acetabular dysplasia patients who underwent CPO, 72.1% participated in sports activities post-operatively, representing an increase compared to pre-operative participation in sports activities. Furthermore, patients post-operatively participated not only in low-impact sport activities, but also in high-impact sports activities. Patients who participated in sports activities post-operatively were allowed to return to FWB faster than those who did not. Therefore, these findings might be useful for advising patients who are concerned about participating in sports activities after CPO.

## Supplementary information

**Additional file 1.** Study questionnaire. Description of data: The questionnaire used for this study was developed specifically for this study. This questionnaire examined patients’ participation in sports activities pre- and post-operatively, patients’ current satisfaction with daily life and sports activities, and related patient-reported outcomes.

## Data Availability

The datasets used and/or analysed during the current study are available from the corresponding author on reasonable request.

## Abbreviations

BMI: Body mass index
CPO: Curved periacetabular osteotomy
FWB: Full weight bearing
HHS: Harris hip score
JHEQ: Japanese Orthopaedic Association Hip-Disease Evaluation Questionnaire
OA: Osteoarthritis
PAO: Periacetabular osteotomy
SLR: Straight leg-raising exercise
VAS: Visual analogue scale

## References

[1] Jingushi S, Ohfuji S, Sofue M, Hirota Y, Itoman M, Matsumoto T, Hamada Y, Shindo H, Takatori Y, Yamada H, Yasunaga Y, Ito H, Mori S, Owan I, Fujii G, Ohashi H, Iwamoto Y, Miyanishi K, Iga T, Takahira N, Sugimori T, Sugiyama H, Okano K, Karita T, Ando K, Hamaki T, Hirayama T, Iwata K, Nakasone S, Matsuura M, Mawatari T (2010). Multiinstitutional epidemiological study regarding osteoarthritis of the hip in Japan. J Orthop Sci.

[2] Murphy SB, Kijewski PK, Millis MB, Harless A (1990). Acetabular dysplasia in the adolescent and young adult. Clin Orthop Relat Res.

[3] Maeyama A, Naito M, Moriyama S, Yoshimura I (2008). Evaluation of dynamic instability of the dysplastic hip with use of triaxial accelerometry. J Bone Joint Surg Am.

[4] Iidaka T, Muraki S, Akune T, Oka H, Kodama R, Tanaka S, Kawaguchi H, Nakamura K, Yoshimura N (2016). Prevalence of radiographic hip osteoarthritis and its association with hip pain in Japanese men and women: the ROAD study. Osteoarthr Cartil.

[5] Novais EN, Heyworth B, Murray K, Johnson VM, Kim YJ, Millis MB (2013). Physical activity level improves after periacetabular osteotomy for the treatment of symptomatic hip dysplasia. Clin Orthop Relat Res.

[6] Bogunovic L, Hunt D, Prather H, Schoenecker PL, Clohisy JC (2014). Activity tolerance after periacetabular osteotomy. Am J Sports Med.

[7] Steppacher SD, Tannast M, Ganz R, Siebenrock KA (2008). Mean 20-year followup of Bernese periacetabular osteotomy. Clin Orthop Relat Res.

[8] van Bergayk AB, Garbuz DS (2002). Quality of life and sports-specific outcomes after Bernese periacetabular osteotomy. J Bone Joint Surg (Br).

[9] Hara D, Hamai S, Fukushi JI, Kawaguchi KI, Motomura G, Ikemura S, Komiyama K, Nakashima Y (2017). Does participation in sports affect osteoarthritic progression after periacetabular osteotomy?. Am J Sports Med.

[10] Naito M, Shiramizu K, Akiyoshi Y, Ezoe M, Nakamura Y (2005). Curved periacetabular osteotomy for treatment of dysplastic hip. Clin Orthop Relat Res.

[11] Karashima H, Naito M, Shiramizu K, Kiyama T, Maeyama A (2011). A periacetabular osteotomy for the treatment of severe dysplastic hips. Clin Orthop Relat Res.

[12] Ezoe M, Naito M, Asayama I (2006). Muscle strength improves after abductor-sparing periacetabular osteotomy. Clin Orthop Relat Res.

[13] Maeyama A, Naito M, Moriyama S, Yoshimura I (2009). Periacetabular osteotomy reduces the dynamic instability of dysplastic hips. J Bone Joint Surg (Br).

[14] Kuroda D, Maeyama A, Naito M, Moriyama S, Yoshimura I, Nakamura Y, Kiyama T (2013). Dynamic hip stability, strength and pain before and after hip abductor strengthening exercises for patients with dysplastic hips. Isokinet Exerc Sci.

[15] Klein GR, Levine BR, Hozack WJ, Strauss EJ, D'Antonio JA, Macaulay W, Di Cesare PE (2007). Return to athletic activity after total hip arthroplasty. Consensus guidelines based on a survey of the hip society and American Association of hip and Knee Surgeons. J Arthroplast.

[16] Behrend H, Giesinger K, Giesinger JM, Kuster MS (2012). The "forgotten joint" as the ultimate goal in joint arthroplasty: validation of a new patient-reported outcome measure. J Arthroplast.

[17] Matsumoto T, Kaneuji A, Hiejima Y, Sugiyama H, Akiyama H, Atsumi T, Ishii M, Izumi K, Ichiseki T, Ito H, Okawa T, Ohzono K, Otsuka H, Kishida S, Kobayashi S, Sawaguchi T, Sugano N, Nakajima I, Nakamura S, Hasegawa Y, Fukuda K, Fujii G, Mawatari T, Mori S, Yasunaga Y, Yamaguchi M (2012). Japanese Orthopaedic Association Hip Disease Evaluation Questionnaire (JHEQ): a patient-based evaluation tool for hip-joint disease. The Subcommittee on Hip Disease Evaluation of the Clinical Outcome Committee of the Japanese Orthopaedic Association. J Orthop Sci.

[18] Harris WH (1969). Traumatic arthritis of the hip after dislocation and acetabular fractures: treatment by mold arthroplasty. An end-result study using a new method of result evaluation. J Bone Joint Surg Am.

[19] Ollivier M, Frey S, Parratte S, Flecher X, Argenson JN (2014). Pre-operative function, motivation and duration of symptoms predict sporting participation after total hip replacement. Bone Joint J.

[20] Wylde V, Blom A, Dieppe P, Hewlett S, Learmonth I (2008). Return to sport after joint replacement. J Bone Joint Surg (Br).

[21] Huch K, Müller KA, Stürmer T, Brenner H, Puhl W, Günther KP (2005). Sports activities 5 years after total knee or hip arthroplasty: the Ulm osteoarthritis study. Ann Rheum Dis.

[22] Steppacher SD, Zurmühle CA, Puls M, Siebenrock KA, Millis MB, Kim YJ, Tannast M (2015). Periacetabular osteotomy restores the typically excessive range of motion in dysplastic hips with a spherical head. Clin Orthop Relat Res.

[23] Ito H, Tanino H, Sato T, Nishida Y, Matsuno T (2014). Early weight-bearing after periacetabular osteotomy leads to a high incidence of postoperative pelvic fractures. BMC Musculoskelet Disord.

[24] Espinosa N, Strassberg J, Belzile EL, Millis MB, Kim YJ (2008). Extraarticular fractures after periacetabular osteotomy. Clin Orthop Relat Res.

