# Supplementary material for: Sports activity participation after curved periacetabular osteotomy for acetabular dysplasia
Source: BMC Musculoskelet Disord. 2020 Sep 28;21:637. doi: 10.1186/s12891-020-03625-3 (PMC7520950; doi:10.1186/s12891-020-03625-3)
Supplement: Supplementary file 1 — Additional file 1. Study questionnaire. Description of data: The questionnaire used for this study was developed specifically for this study. This questionnaire examined patients’ participation in sports activities pre- and post-operatively, patients’ current satisfaction with daily life and sports activities, and related patient-reported outcomes. [file 12891_2020_3625_MOESM1_ESM.docx]

**Questionnaire on sports activities after curved periacetabular osteotomy**

Please read the enclosed instructions before you fill out this questionnaire. Please provide responses in the blank spaces provided and circle the items in parentheses that apply to you. This questionnaire is 10 pages long and should take about 10 minutes to complete. The term “sports activities” refers to any exercise performed voluntarily. If you have had multiple surgeries, please provide answers with reference to the last surgery you had. If you agree to the secondary use of your data, please check the box below to provide consent.

We apologize for the busy schedule but sincerely appreciate your cooperation.

□ I agree to the secondary use of my data.

Date / /

**[Basic information]**

1. Name:

2. Age:

3. Sex: ( Male / Female )

4. Side of osteoarthritis: ( Right / Left / Both )

Side of operation: ( Right / Left / Both )

5. Date of operation: / /

6. Date of discharge: / /

**[Information before surgery]**

7. Did you use a walking aid (for example, a cane or walker) to walk? If you used different aids indoors and outdoors, please describe both.

Indoor No walking aid / Cane ( T-cane / Lofstrand crutch / Crutch /

Other ) / Wheeled walker / Other

Outdoor No walking aid / Cane ( T-cane / Lofstrand crutch / Crutch /

Other ) / Wheeled walker / Other

8. Did you participate in sports activities before surgery? ( Yes / No )

**If you answered “yes” to Question 8, please answer Questions 9 and 10; otherwise, please skip to Question 11.**

9. Please circle the sports activities that you participated in (circle all that apply).

Walking / Training / Calisthenics / Jogging / Road cycling

Aerobics / Pilates / Badminton / Yoga / Golf / Ballet

Swimming / Bowling / Climbing / Table tennis / Skiing

Single tennis / Double tennis / Snow board / Horse-riding

Volleyball / Track and field / Other

10. How often did you participate in sports activities? (if you participated in more than one, please choose the one you performed most often)

Frequency: times/month, times/week

Period: From / / to / /

Type:

**[Information after surgery]**

11. Did you use a walking aid (for example, a cane or walker) when you were discharged? If you used different aids indoors and outdoors, please describe both.

Indoor No walking aid / Cane ( T-cane / Lofstrand crutch / Crutch /

Other ) / Wheeled walker / Other

Outdoor No walking aid / Cane ( T-cane / Lofstrand crutch / Crutch /

Other ) / Wheeled walker / Other

12. When did you stop using a walking aid?

Indoor months weeks after surgery / Still using

Outdoor months weeks after surgery / Still using

13. Did you participate in sports activities after surgery? ( Yes / No )

**If you answered “yes” to Question 13, please answer Questions 14–17; otherwise please skip to Question 18.**

14. Please circle the sports activities that you participate in (circle all that apply).

Walking / Training / Calisthenics / Jogging / Road cycling

Aerobics / Pilates / Badminton / Yoga / Golf / Ballet

Swimming / Bowling / Climbing / Table tennis / Skiing

Singles tennis / Doubles tennis / Snowboard / Horse riding

Volleyball / Track and field / Other

15. How often do you participate in sports activities? (if you participate in more than one, please choose the one you perform most often)

Frequency: times/month, times/week

Period: From / / to / /

Type:

16. When did you start participating in sports activities after surgery?

years and months after surgery

17. Why did you choose to participate in sports activities? (circle all that apply)

No pain / Moving as patients want / Liking exercise / Healthy

Physical strength or maintenance / Lack of exercise

Enjoying or distraction / Prevention of obesity

Other:

**If you answered “no” to Question 13, please answer Questions 18–20; otherwise please skip to Question 21.**

18. Why did you *not* participate in sports activities? (circle all that apply)

Pain / Unable to move as desired / Advice from doctor

No confidence / Fear of damage to the hip

Other:

19. Do you hope to participate in sports activities in the future? ( Yes / No )

20. If you answered “yes” to Question 19, please choose the type(s) of sport activities that you would like to participate in (circle all that apply).

Walking / Training / Calisthenics / Jogging / Road cycling

Aerobics / Pilates / Badminton / Yoga / Golf / Ballet

Swimming / Bowling / Climbing / Table tennis / Skiing

Singles tennis / Doubles tennis / Snow board / Horse-riding

Volleyball / Track and field / Other

**[Current information]**

21. Please indicate your current satisfaction with daily activities and sports activities.

|  |  | Extremely dissatisfied | Slightly dissatisfied | Neither | Slightly satisfied | Very satisfied |
| --- | --- | --- | --- | --- | --- | --- |
|  | Satisfaction with daily activities | 1 | 2 | 3 | 4 | 5 |
|  | Satisfaction with sports activities | 1 | 2 | 3 | 4 | 5 |

22. How aware you are of your hip joint in everyday life? Please select one answer for each item by ticking the appropriate box.

|  |  | Never | Almost never | Seldom | Sometimes | Mostly |
| --- | --- | --- | --- | --- | --- | --- |
| 1. | In bed at night | □ | □ | □ | □ | □ |
| 2. | When you are sitting on a chair for more than 1 hour | □ | □ | □ | □ | □ |
| 3. | When you are walking for more than 15 minutes | □ | □ | □ | □ | □ |
| 4. | When you are taking a bath/shower | □ | □ | □ | □ | □ |
| 5. | When you are traveling in a car | □ | □ | □ | □ | □ |
| 6. | When you are climbing stairs | □ | □ | □ | □ | □ |
| 7. | When you are walking on uneven ground | □ | □ | □ | □ | □ |
| 8. | When you are standing up from a low-sitting position | □ | □ | □ | □ | □ |
| 9. | When you are standing for long periods of time | □ | □ | □ | □ | □ |
| 10. | When you are doing housework or gardening | □ | □ | □ | □ | □ |
| 11. | When you are taking a walk/hiking | □ | □ | □ | □ | □ |
| 12. | When you are doing your favorite sport | □ | □ | □ | □ | □ |

This concludes the survey.

**Thank you very much for your responses!**

Please send your completed questionnaire in the return envelope provided.

If you have any comments or questions about this survey, please write them in the box below.

Thank you for your cooperation.
